# Regular Aerobic Training Combined with Range of Motion Exercises in Juvenile Idiopathic Arthritis

**DOI:** 10.1155/2014/748972

**Published:** 2014-01-22

**Authors:** Mine Doğru Apti, Özgür Kasapçopur, Murat Mengi, Gülnur Öztürk, Gökhan Metin

**Affiliations:** ^1^Department of Sports Medicine, Istanbul Faculty of Medicine, Istanbul University, 34093 Istanbul, Turkey; ^2^Department of Pediatric Rheumatology, Cerrahpasa Faculty of Medicine, Istanbul University, 34098 Istanbul, Turkey; ^3^Department of Physiology, Cerrahpasa Faculty of Medicine, Istanbul University, 34098 Istanbul, Turkey; ^4^Department of Physiotherapy and Rehabilitation, Faculty of Health Sciences, Trakya University, 22030 Edirne, Turkey

## Abstract

*Objective*. To assess the effects of regular aerobic training combined with range of motion (ROM) exercises on aerobic capacity, quality of life, and function in children with juvenile idiopathic arthritis (JIA). *Methods*. Thirty patients with JIA and 20 healthy age-matched controls (mean age ± SD, 11.3 ± 2.4 versus 11.0 ± 2.3, resp.; *P* > 0.05) were included. All patients performed aerobic walking (4 days a week for 8 weeks) and active and passive ROM exercises of involved joints. All patients completed the childhood health assessment questionnaire (CHAQ) and the child health questionnaire. ROM measurements of joints were performed by using universal goniometer. Aerobic capacity was determined by measuring peak oxygen uptake (VO_2peak_) during an incremental treadmill test. *Results*. Peak oxygen uptake and exercise duration were significantly lower in JIA group than in controls (32.5 ± 6.6 versus 35.9 ± 5.8 and 13.9 ± 1.9 versus 15.0 ± 2.0, resp.; *P* < 0.05 for both). Eight-week combined exercise program significantly improved exercise parameters of JIA patients (baseline versus postexercise VO_2peak_ and exercise duration, 32.5 ± 6.6 to 35.3 ± 7.9 and 13.9 ± 1.9 to 16.3 ± 2.2, resp.; *P* < 0.001 for both). Exercise intervention significantly improved CHAQ scores in JIA patients (0.77 ± 0.61 to 0.20 ± 0.28, *P* < 0.001). *Conclusion*. We suggest that regular aerobic exercise combined with ROM exercises may be an important part of treatment in patients with JIA.

## 1. Introduction

Juvenile idiopathic arthritis (JIA) usually occurs before 16 years of age and mainly affects peripheral joints leading to pathological changes [1, 2]. Therefore, the importance of starting and maintaining therapeutic exercises in early periods in order to improve range of motion (ROM) in arthritic joints is constantly stressed [3–5]. However, daily activity level and cardiopulmonary capacity of patients with JIA are significantly lower when compared to healthy peers [6–10]. Interestingly, lower physical capacity in children with JIA was shown to be unrelated to disease status whether active or in remission [8]. Besides reduced cardiopulmonary capacity, forced adjourning physical functions during active disease state are an important factor among the reasons of inactivity. In addition, lack of adequate knowledge on the benefits of exercise and considering exercise as harmful to joints result in self-limitation even during remission period [11, 12]. Furthermore, it was suggested that fear and social isolation seen in chronic diseases might lead to inactivity in JIA patients [13].

There are conflicting results from recent studies that investigated the effects of exercise programs on cardiopulmonary capacity in this group of patients. Some studies reported beneficial effects of exercise on joint ROMs and cardiopulmonary and functional capacities [14–17], whereas, at a lesser extent, several studies reported opposite results [18, 19]; thus there is no consensus on this subject. Previous studies also differ in terms of duration, frequency, and content of exercise programs applied to patients with JIA [14, 16–21]. In this study, we aimed to determine aerobic capacities of children with JIA by using exercise tests. And also we aimed to investigate the effects of regular aerobic training combined with active and passive exercises addressing functional impairment resulting both systemic effects of the disease and joint involvement.

## 2. Methods

### 2.1. Subjects

Forty-seven JIA patients (M/F, 18/29; age range, 8–16 years) were included in the study. Twenty healthy children (M/F, 9/11; age range, 9–13 yr) served as controls. Prior to testing, parents of subjects read and signed a consent form that was approved by the University's Policy and Review Committee on Human Research. Controls had a sedentary lifestyle and did not participate in any regular sportive activities. Patients were diagnosed by a pediatric rheumatologist (ÖK) according to the criteria of International League Against Rheumatology [22]. Patient group consisted of systemic (*n* = 2), polyarticular (*n* = 24), oligoarticular (*n* = 16), enthesitis related arthritis (*n* = 1), and psoriatic (*n* = 4) types of JIA. The Turkish version of childhood health assessment questionnaire (CHAQ) and the child health questionnaire [23] were completed by the parents or the guards of children after giving information and answering the questions raised by parents. Fourteen patients declined to undergo exercise program and withdrew from the study, 2 patients were lost to follow-up, and 1 patient was dropped due to hospitalization for other reasons. At the end, 30 patients completed the whole study protocol and were included in final statistical analyses.

### 2.2. Anthropometry and ROM Measurements

Body mass was measured on a balance beam medical scale (Fairbanks) to the nearest 0.1 kg. Stature was measured on a stadiometer (Holtain, UK) to an accuracy of ±0.5 cm with the subject barefoot, feet together, and head level. ROM values in shoulder, elbow, wrist, hip, knee, and ankle joints were measured; triple and average results were recorded. For normal ROM values of joints, Kendall-McCreary norms were used [24].

### 2.3. Experimental Protocol

The peak oxygen uptakes (VO_2peak_) of all subjects were measured during incremental exercise testing. Each child first underwent a comprehensive physical examination which included a 12-lead electrocardiogram (ECG) recording and blood pressure measurement at rest. Prior to exercise tests, all participants underwent pulmonary function tests (Ocean Win Spiro 2.36 B software with Spirobank; Medical International Research, Italy). All tests were performed in an air-conditioned laboratory room at 20–22°C and 40% relative humidity of air, to minimize thermal stress. Subjects had a light breakfast 2 hours before exercise and abstained from strenuous exercise for a week prior to test protocol.

### 2.4. Cardiopulmonary Exercise Testing (CPET)

All the subjects exercised on a motorized treadmill ergometer. A modified Bruce exercise protocol [25] was used with an additional stage 0 (3 min; speed, 1.7 mph; 5% gradient). The treadmill protocol is a commonly used exercise test to assess exercise capacity and the electrocardiographic response to exercise stress in children and adults. The test protocol is suitable for stressing healthy subjects and patients [26, 27]. Each test was terminated by subject fatigue or maximal exercise level. It was considered maximal level if the subject achieved two of the three following test criteria: (1) the plateau of oxygen uptake with increasing work load, (2) a respiratory exchange ratio (RER) of 1.00 or higher, and (3) heart rate reaching 85% of age-predicted maximal heart rate (Max HR). This parameter was calculated by subtracting subject's age from 220 (Max HR = 220 − age). During exercise test, a full 10-electrode, 12-lead ECG was monitored. In all cases, a Quinton 5000 recorder and lead system (Quinton Instrument Company, Seattle, USA) were utilized to monitor and record ECG. Heart rate was monitored from ECG. Blood pressure was measured every three minutes using cuff manometer for children (ERKA, Germany).

### 2.5. Direct Measurements of Peak Oxygen Uptake

Ventilation (VE), oxygen uptake (VO_2_), and carbon dioxide production (VCO_2_) were monitored continuously, breath-by-breath, at rest, during exercise, and for 3 minutes of recovery after exercise, using a CPET system (Cortex MetaLyzer 3B, Cortex Biophysik GmbH, Leipzig, Germany). The system was calibrated before each test with standard gases of known O_2_ and CO_2_ concentrations. The most elevated VO_2_ measured over 30 seconds during the last period of the exercise was considered as the VO_2peak_. RER was calculated as VCO_2_/VO_2_.

### 2.6. Exercise Training Program

Patients performed aerobic type walking (4 days a week) and active and passive ROM exercises (10–15 repeat/set, 2 sets/day, 7 days/week) in combination for 8 weeks. Program was started under supervision of specialists (MDA, MM) with 30 minutes walking during the first 2 weeks. Then, the exercise duration was incremented 5 minutes for each week. Exercise intensity adjustment was based on anaerobic threshold (AT) determined by CPET for each patient. Before and after aerobic exercise, stretching exercises were performed for warming up and cooling down.

### 2.7. Statistical Analysis

In all comparisons *P* values < 0.05 were considered statistically significant. Physical characteristics and CPET results of JIA and control groups were compared using “independent samples *t*-test” (Table 1). Changes in CHAQ scores and CPET results (Table 2), as well as goniometric measurements (Table 3) before and after exercise program were compared by using the paired samples *t*-test.

## 3. Results

JIA and control groups were age matched. However, there were significant differences in the mean weight and height values between the two groups (Table 1). In comparison of cardiopulmonary exercise test results, there were statistically significant differences in VO_2peak_, VE_peak_, RER, maximal heart rate, % predicted heart rate, exercise duration, and VO_2AT_ between the patient and control groups (Table 1). To determine the effects of exercise program, we compared exercise parameters before and after completion of exercise program. There were significant differences in VO_2peak_, VE_peak_, RER, maximal heart rate, % predicted heart rate, % predicted VO_2_, exercise duration, AT VO_2_, resting heart rate, and resting SBP (Table 2).

Comparison of ROM values before and after exercise program showed that shoulder abduction and flexion, wrist flexion and extension, elbow flexion, hip flexion, knee flexion and extension, and ankle plantar flexion and dorsiflexion were significantly changed in both extremities (Table 3).

## 4. Discussion

Juvenile idiopathic arthritis patients were consistently found to have lower cardiopulmonary exercise capacity compared to healthy peers. In a meta-analysis of 144 patients from 16 different studies, the mean VO_2peak_ was found to be 21.8% lower in JIA patients than in healthy controls [6]. Metin et al. [8], reported significant reductions of VO_2peak_ and %predicted VO_2_ in 34 patients with JIA with respect to controls. Similarly, van Brussel et al. [9] showed that the mean VO_2peak_ values of 62 JIA patients were significantly lower than control subjects. In addition, Lelieveld et al. [7] found lower cardiopulmonary capacities in JIA patients when compared to healthy peers.

In our study (Table 1), exercise duration of JIA group, VO_2peak_, VO_2AT_, VE_peak_, RER, Max HR, and % predicted HR values were significantly lower compared to healthy controls. Therefore, our results were in accordance with the previous data mentioned in the former paragraph [6–9].

As known, in polyarticular juvenile idiopathic arthritis (PJIA) five or more joints are affected within the first 6 months of disease occurrence. Thus, joint destruction, ankylosis, and ROM limitation in PJIA patients start earlier with respect to oligoarticular juvenile idiopathic arthritis (OJIA) and progress during the course of disease [1, 28]. In several studies, age of disease onset and number of affected joints were reported as the most important factors playing role in development of functional limitation in the long term [29–31].

In a study of healthy children, physical activity and peak VO_2_ showed poor correlation [32]. In addition, healthy children may exert a small improvement in peak VO_2_ by increasing physical activity (ceiling effect) [33, 34]. However, Takken et al. [35], demonstrated significant correlation between the daily physical activity and peak VO_2_ in patients with JIA which was different from healthy children. These authors suggested that children with low physical capacity due to chronic disease may not be limited by the ceiling effect and increasing physical activity may contribute to development of peak VO_2_ [35].

In our study, examination of cardiopulmonary exercise tests of the group having exercise program showed significant differences in metabolic parameters after the program with respect to basal values. These changes were significant in % predicted VO_2_ (*P* < 0.05), moderately significant in VO_2peak_, RER, VO_2AT_, resting heart rate, and resting systolic blood pressure (*P* < 0.01) and highly significant in VE_peak_, Max HR, % predicted HR, and exercise duration (*P* < 0.001) (Table 2).

Recently, the first randomized, controlled trial of Takken et al. [18] reported that, following an exercise program CHAQ score, VO_2peak_ and distance of a six-minute walk test were similar between exercise and control groups. Singh-Grewal et al. [19] reported similar significant improvements in CHAQ scores of high intensity (dance, cardio, and karate) and low intensity (Qi-Gong-Tai Chi, etc.) exercise groups, whereas VO_2peak_ showed no change. Similarly, Epps et al. [20] reported significant improvements in CHAQ scores and % predicted HR values of combined (water and land exercise) or land exercise program groups. Exercise duration showed no difference. Recently, our group showed that a land-based home exercise program improved both physical function and quality of life in patients with JIA [36]. Besides physical disability, psychological alterations or psychiatric conditions such as depression may occur during the course of this condition [37]. This may also affect the quality of life.

In our study, exercise group showed significant improvement in CHAQ scores (Table 2) which is in parallel with previous studies [19, 20]. On the other hand, JIA patients who underwent an exercise program showed improvements in VO_2peak_ and exercise duration and these results are not in accordance with the above-mentioned three studies [18–20]. In another study conducted via the Internet, patients were encouraged to exercise and their exercise duration was significantly increased [15]. Furthermore, Moncur et al. [16], reported significant increases in VO_2peak_ and exercise duration after a regular exercise program. Klepper [14] reported significant increase in the distance taken in a 9-minute walk test after a planned exercise program.

Although frequency and intensity may differ in different subgroups, it has been well known that JIA patients may show ROM limitations due to joint destruction, synovial effusion, and muscle spasm. Besides, studies reporting the effects of exercise programs addressing ROM limitations are very limited, although these exercises are used widely in management of JIA patients. Bacon et al. [17] reported increased hip mobility of children with JIA after water exercises. Epps et al. [20] reported decrease in number of limited joints but the difference failed to reach statistically significance level. Similarly, Takken et al. [18] found that joint ROM values did not differ before and after an exercise program. Exercise programs of those three studies [17, 18, 20] and our exercise program were different. Previous researchers used exercise programs addressing cardiopulmonary capacity rather than joint mobility. They did not include therapeutic programs addressing affected joints.

There are several limitations to this study that deserve comment. A control group from JIA patients without exercise intervention could improve the validity of our results. On the other hand, we felt that it might be ethically problematic not to apply exercise program, although we knew that it was useful.

We combined ROM exercises and aerobic program which significantly improved shoulder abduction and flexion of both extremity, wrist flexion and extension, elbow flexion, hip flexion, knee flexion and extension, and plantar flexion and dorsiflexion in JIA patients (Table 3). In addition, we also found elbow extension and hip external rotation on the right side, and hip internal rotation on the left side reached physiological limits. Cardiopulmonary capacity in our JIA group was lower than healthy peers. With the aid of exercise program, their cardiopulmonary capacity was also increased (Table 2). In addition, changes in CHAQ scores and ROM levels suggest that exercise programs also affect joint involvement and functional status (Tables 2 and 3).

## 5. Conclusion

We suggest that combination of ROM exercise and regular aerobic exercise in JIA treatment may offer important benefits and should be prescribed as an additional module. Physicians should discuss with and give adequate information to family members of JIA patients. Patient's interest, cardiopulmonary capacity, and joint involvement should also be considered.

## Figures and Tables

**Table 1:** Physical characteristics and CPET results of JIA and control groups.

	JIA (*n* = 30)	Control (*n* = 20)	*P* value
Age, year	11.37 ± 2.48	11.04 ± 2.33	N.S.
Height, cm	136.91 ± 13.96	148.05 ± 13.36	0.004
Weight, kg	35.43 ± 12.06	44.85 ± 14.85	0.008
Rest HR, bpm	96.70 ± 17.31	91.20 ± 11.56	N.S.
Rest VE, L/min	6.12 ± 0.94	5.78 ± 0.85	N.S.
HR_AT_, bpm	159.42 ± 16.37	166.11 ± 14.77	N.S.
VO_2AT_, mL/kg/min	26.71 ± 5.91	31.64 ± 8.28	0.050
Max HR, bpm	179.50 ± 14.68	187.8 ± 12.09	0.008
% Predicted HR	85.50 ± 8.13	90.65 ± 7.63	0.007
VO_2peak_, mL/kg/min	32.51 ± 6.67	35.95 ± 5.87	0.050
Predicted VO_2_, %	67.62 ± 18.33	73.49 ± 17.08	N.S.
VE_peak_, L/min	37.75 ± 10.50	46.25 ± 12.95	0.002
RER	0.98 ± 0.10	0.93 ± 0.70	0.050
Exercise time, min	13.93 ± 1.95	15.00 ± 2.04	0.047

Abbreviations: HR: heart rate; VE: minute ventilation; AT: anaerobic threshold; Max: maximal; RER: respiratory exchange ratio; JIA: juvenile idiopathic arthritis; NS: not significant; CPET: cardiopulmonary exercise test.

**Table 2:** CHAQ scores and CPET results of the patients before and after an 8-week exercise program.

	Before (*n* = 30)	After (*n* = 30)	*P* value
CHAQ score	0.77 ± 0.61	0.20 ± 0.28	0.001
Rest HR, bpm	96.70 ± 17.31	86.96 ± 12.62	0.002
Rest SBP, mmHg	114.66 ± 14.52	105.00 ± 11.75	0.002
Rest DBP, mmHg	66.66 ± 10.33	65.73 ± 7.98	N.S.
Rest VE, L/min	6.12 ± 0.94	6.11 ± 1.03	N.S.
HR_AT_, bpm	159.42 ± 16.37	164.19 ± 12.73	N.S.
VO_2AT_, mL/kg/min	26.71 ± 5.91	29.85 ± 7.52	0.003
Max HR, bpm	179.50 ± 14.68	187.20 ± 11.01	0.001
% Predicted HR	85.50 ± 8.13	90.60 ± 6.78	0.001
Max SBP, mmHg	149.23 ± 22.43	145.93 ± 20.62	N.S.
Max DBP, mmHg	73.23 ± 24.54	67.96 ± 12.73	N.S.
VO_2peak_, mL/kg/min	32.51 ± 6.67	35.33 ± 7.94	0.001
% Predicted VO_2_	67.62 ± 18.33	70.35 ± 16.79	0.017
VE_peak_, L/min	37.75 ± 10.50	45.33 ± 12.63	0.001
RER	0.98 ± 0.10	1.07 ± 0.10	0.002
Exercise time, min	13.93 ± 1.95	16.3 ± 2.23	0.001

Abbreviations: HR: heart rate; VE: minute ventilation; AT: anaerobic threshold; Max: maximal; RER: respiratory exchange ratio; SBP: systolic blood pressure; DBP: diastolic blood pressure; NS: not significant.

**Table 3:** Goniometric measurements of the patients before and after an 8-week exercise program.

	Before (*n* = 30)	After (*n* = 30)	*P* value
Shoulder abduction			
Right	177.97 ± 4.32	179.45 ± 2.15	0.023
Left	178.77 ± 3.03	179.84 ± 0.89	0.041
Shoulder flexion			
Right	178.19 ± 4.02	179.84 ± 0.89	0.021
Left	177.84 ± 5.24	180.00 ± 0.00	0.029
Elbow flexion			
Right	143.65 ± 7.60	148.35 ± 2.93	0.000
Left	141.65 ± 10.3	148.42 ± 3.38	0.001
Wrist flexion			
Right	81.23 ± 9.67	87.90 ± 7.27	0.000
Left	80.71 ± 11.22	88.23 ± 4.57	0.000
Wrist extension			
Right	62.48 ± 13.02	68.71 ± 3.64	0.004
Left	61.68 ± 10.64	67.90 ± 4.96	0.001
Hip flexion			
Right	122.61 ± 4.86	124.87 ± 1.11	0.008
Left	122.32 ± 4.42	124.84 ± 0.89	0.002
Knee flexion			
Right	134.52 ± 6.96	138.97 ± 2.30	0.001
Left	130.90 ± 10.76	139.03 ± 2.81	0.000
Knee extension			
Right	1.42 ± 2.86	0.32 ± 1.79	0.016
Left	1.35 ± 2.79	0.30 ± 1.64	0.018
Ankle dorsiflexion			
Right	17.68 ± 4.02	19.68 ± 1.24	0.004
Left	17.90 ± 3.60	20.06 ± 0.02	0.003
Ankle plantar flexion			
Right	33.94 ± 8.25	42.42 ± 5.72	0.000
Left	35.68 ± 9.19	43.68 ± 4.15	0.000
